# Evaluation of the Use of Platelet-Rich Fibrin Xenologous Membranes Derived from Bubaline Blood in Canine Periodontal Defects

**DOI:** 10.3390/vetsci8100210

**Published:** 2021-09-28

**Authors:** Poranee Banyatworakul, Thanaphum Osathanon, Chanin Kalpravidh, Prasit Pavasant, Nopadon Pirarat

**Affiliations:** 1 Dental Stem Cell Biology Research Unit, Faculty of Dentistry, Chulalongkorn University, Bangkok 10330, Thailand; Poranee.ba@student.chula.ac.th; 2 Department of Pathology, Faculty of Veterinary Science, Chulalongkorn University, Bangkok 10330, Thailand; 3 Department of Anatomy, Faculty of Dentistry, Chulalongkorn University, Bangkok 10330, Thailand; prasit.pav@chula.ac.th; 4 Department of Surgery, Faculty of Veterinary Science, Chulalongkorn University, Bangkok 10330, Thailand; chanin.k@chula.ac.th; 5 Wildlife Exotic and Aquatic Pathology-Research Unit, Department of Pathology, Faculty of Veterinary Science, Chulalongkorn University, Bangkok 10330, Thailand

**Keywords:** platelet-rich fibrin, periodontal, healing, bubaline, canine

## Abstract

Periodontal disease is the most common oral disease in dogs. Platelet-rich fibrin (PRF) is widely utilized to facilitate soft and hard tissue healing and has been proposed in periodontal healing in small animal treatment. However, the quality and amount of autologous PRF is compromised in animals with systemic diseases. The present study aimed to evaluate the efficacy of xenologous bubaline blood-derived PRF (bPRF) on periodontal tissue healing in canine periodontal defects. Split-mouth design was employed in twenty dogs diagnosed with periodontal disease. The defects were divided randomly into two groups: the open-flap debridement (OFD)-treated group and the OFD with bPRF (OFD+bPRF) application group. Results demonstrated that gingival index and periodontal probing depth decreased significantly in the OFD+bPRF group compared with those treated with OFD alone. Application of bPRF in periodontal defects also promoted fibrous tissue formation, as confirmed by the marked increase in fibrosis score. bPRF application significantly increased *COL1A1* and *PDGFB* mRNA levels at day 14 compared with the baseline. Taking this evidence together, bPRF provided a favorable therapeutic modality in canine periodontal defects. bPRF could be an alternative biomaterial for the treatment of periodontal defects in dogs.

## 1. Introduction

Periodontal disease (PD) constitutes the most common oral problem in companion dogs. The incidence and severity of PD increases significantly with age [1,2]. Although, the etiology of PD is multifactorial, inflammation is a crucial process in the development and progression of periodontal pathogenesis [3]. PD results to the destruction of periodontal tissues (gingiva, cementum, alveolar bone, and periodontal ligament), subsequently leading to tooth mobility and tooth loss. A common PD prevention is good oral hygiene practice to reduce the accumulation of dental plaque. The conventional treatment in companion dogs consists of professional dental cleaning and root planing in combination with periodontal surgery to control disease progression. Several attempts of periodontal regeneration treatment focused on minimizing the inflammatory process and accelerating wound healing [4,5,6].

Periodontal healing can lead to both periodontal tissue repair and regeneration [7]. In repair healing, the periodontium does not fully restore the normal structure and function. However, periodontal regeneration involves the regeneration of several tissues, including alveolar bone, gingiva, cementum, and periodontal ligament. These regenerated tissues should exhibit the normal form and properly function in a physiological context. Hence, the ultimate goal is to obtain regeneration healing. 

Platelet concentrates and related products have been developed and employed in periodontal clinical application for decades [8]. Platelet-rich fibrin (PRF) is a second-generation platelet concentrate consisting of a fibrin matrix with entrapped platelets, leukocytes, and numerous growth and differentiation factors. The exemplification of active growth factors found in PRF includes platelet-derived growth factor (PDGF), transforming growth factor ß_1_ (TGF-ß_1_), vascular endothelial growth factor (VEGF), insulin-like growth factor 1 (IGF-1), fibroblast growth factor (FGF), and epidermal growth factor (EGF) [9]. These growth factors exhibit crucial roles in periodontal healing and regeneration [10].

PRF is considered as a therapeutic tool to reduce pain and inflammation in oral wounds [9,11,12]. Autologous PRF also promotes bone regeneration [13,14,15,16,17]. Further, autologous PRF stimulates soft and hard tissue healing and regeneration in post-extraction alveolar sockets in dogs with spontaneous periodontal disease [18]. Our previous study on companion dogs demonstrates a favorable response of autologous PRF in canine periodontal regeneration [19]. The combination of PRF with autologous bone results in the increase in osteointegration of dental implants [20]. From systematic review and meta-analysis, leukocyte PRF treatment enhances clinical attachment levels compared with coronally advanced flap alone in gingival recession defects [21,22]. Autologous platelet concentrates can be used in combination with open-flap debridement and bone grafting to improve furcation treatment [23].

Host health status is of concern in small dog breeds when obtaining a high volume of autologous blood. Anemia, thrombocytopenia, and platelet dysfunctions limit PRF preparation and quality in small elderly animals. Finding an alternative PRF from either an allogeneic or xenologous source has gained great interest and attention. The quantity and architecture of fibrinogen and thrombin affect the mechanical properties of PRF. It has been reported that bubaline blood demonstrates the highest fibrinogen levels compared with those from human, bovine, and ovine sources [24]. Hence, bubaline blood could be a xenologous candidate source of PRF preparation in small animal treatment. The present study aimed to examine the efficacy of xenologous bubaline blood derived PRF (bPRF) on periodontal tissue healing in canine periodontal defects. 

## 2. Materials and Methods

### 2.1. PRF Preparation 

The study was conducted using bubaline blood. Four Thai buffaloes were recruited for blood collection and PRF preparation. Screening of donor blood was carried out prior to the experiments. To limit the confounding factor, donors were quarantined from severe zoonotic diseases and lived in the same environment. Fresh blood samples (10 mL) were collected from the cephalic vein of Thai swamp buffalo (*Bubalus bubalis var. kerebau*) and transferred to glass tubes without anticoagulants. The samples were centrifuged immediately and kept in 15 mL glass tubes. PRF membranes were produced using a laboratory centrifuge (Hettich EBA 20, Sigma-Aldrich, Burlington, MA, USA) at 857G for 10 min (radius motor = 86 mm) [9], resulting in the formation of a PRF clot. The PRF membrane was separated using scissors and then manually compressed with sterile gauze to drive out the fluid. The serum exudate was eliminated. The serum exudate was collected for preserving the graft material hydration, surgical site rinsing, and PRF storage. The PRF membranes were cut with a biopsy punch to create round pieces with a 6-mm diameter. Punch biopsy sampling was performed at 2 cm above the separation zone.

### 2.2. Clinical Study in Animals

The study design was a prospective randomized controlled trial in companion dogs. Experimental animals were recruited from the Oral Surgery Unit at the Small Animal Teaching Hospital, Faculty of Veterinary Science, Chulalongkorn University with the following inclusion criteria: (1) mesocephalic breed, age 1–5 years, weight 5–15 kg (cephalic index = 1); (2) healthy dogs based on physical examination and blood results; (3) stage 2 of PD on carnassial teeth (maxillary 4th premolars and mandibular 1st molar). Stage 2 periodontal disease refers to periodontitis that exhibits up to 25% of attachment loss, or at most there is a stage 1 furcation involvement in multi-rooted teeth and up to 25% as measured either by probing of the clinical attachment level or radiographic determination of the distance of the alveolar margin from the Cemento-enamel junction (CMJ) relative to the length of the root. The exclusion criteria for selection were: (1) systemic diseases including uncontrollable metabolic disease, cancer, and immunosuppressive disease; (2) dogs receiving oral or parenteral antibiotics and anti-inflammatory drugs within the past 30 days; (3) dogs with periodontal pockets greater than 5 mm in more than 3 teeth per quadrant; (4) dogs with periodontal complications and fractured teeth. Informed consent was obtained from the owners, and the experiments were conducted in accordance with the standard guidelines for animal welfare of experimental animals approved by Chulalongkorn University Animal Care and Use Committee (Animal Use Protocol #1931035). 

Dental assessments and scoring were performed using the Wiggs and Lobprise scoring system [25,26]. A split-mouth clinical study was performed. Forty periodontal defects were included and randomly divided into 2 groups using a list of random assignments generated by a computer. The 108, 208, 309 or 409 were randomly selected for the control or treatment group. Simple randomized methods were employed in the study. In the first group, defects were treated with dental scaling, open-flap debridement, and root planing. This group was assigned as the control group (OFD-treated group, *n* = 20). In another group, defects were treated with dental scaling, root planing, and open-flap debridement as well as bubaline blood-derived PRF application. This second group was identified as the treatment group (OFD+bPRF group, *n* = 20). 

### 2.3. Surgical Procedure and PRF Administration

**Figure 1:**
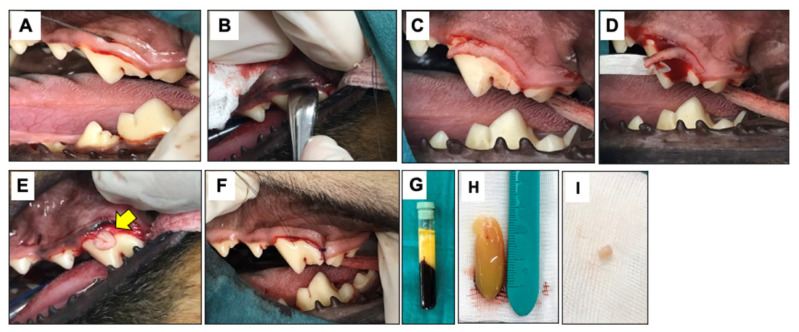
Surgical procedures. The modified Widman flap technique (MWF) is illustrated. An reverse bevel incision was performed (**A**), and subsequently full thickness mobilized mucoperiosteal flap was reflected (**B**). An intrasulcular incision (**C**) and horizontal incision (**D**) were performed. For the OFD+bPRF group, bubaline blood-derived PRF (bPRF) was placed over the defects after open flap (**E**). Yellow arrow indicates bPRF. The surgical area was closed with a simple interdental suture (**F**). bPRF was prepared from fresh blood samples. Blood was collected from Thai buffalo cephalic vein and centrifuged immediately at 3000 rpm for 10 min (**G**). bPRF clot was harvested from between upper layer (acellular plasma) and bottom layer (red corpuscles) (**H**). The bPRF membranes were cut with a biopsy punch to create round pieces with a 6 mm diameter (**I**). OFD; open-flap debridement-treated group, OFD+bPRF; open-flap debridement and bubaline blood-derived platelet-rich fibrin-treated group.

Full mouth radiographic and oral examinations were performed prior to surgery. Animals were given a general physical examination prior to surgery and underwent a premedication of 0.03 mg/kg acepromazine in combination with 0.3 mg/kg morphine via an intramuscular route. Subsequently, 4 mg/kg propofol induction was performed via an intravenous route. Anesthesia was maintained with isoflurane inhalation. Local anesthesia (0.5% bupivacaine) was given to minimize pain. Parenteral antibiotic (25 mg/kg cefazolin) was given as a prophylactic. Chlorhexidine rinsing were carried out after oral examination. Animals in the OFD group received the modified Widman flap (MWF) technique (Figure 1). Briefly, the MWF technique was performed with reverse bevel incision, full thickness mobilized mucoperiosteal flap, intrasulcular incision and horizontal incision along the alveolar crest. The surgical area was closed with the mucoperiosteal flap with 4–0 monofilament absorbable suture materials (PD-X, Kruuse, Langeskov, Denmark). In the OFD+bPRF group, bubaline blood-derived PRF was placed in the defects after open flap, and the PRF membrane was positioned over the root surface underneath the cemento-enamel junction (CEJ). The mucoperiosteal flap closure was placed in the same manner as those performed in the OFD group. For post-operative management, 15 mg/kg amoxycillin-clavulanic acid and 4 mg/kg tramadol hydrochloride were prescribed as prophylactic antibiotics and painkillers for 5 days. Chlorhexidine gluconate (0.12%) rinsing was used as a local antiseptic treatment two times a day for 14 days. Stitch removal was performed on day 14. Soft food was fed for a period of 14 days. 

### 2.4. Clinical Examination

The plaque index (PI), gingival index (GI), and periodontal pocket depth (PPD) were determined under anesthesia in accordance with guidelines previously reported [27,28]. For evaluating the PI, the presence or absence of plaque accumulation on six different parts of the surfaces of each tooth was recorded. The GI was examined in four different parts of the surfaces of the teeth (mesial, distal, buccal, and lingual/palatal sites). The color, density, and consistency of gingiva were examined. The criteria for PI and GI were shown in Table 1. The periodontal pocket depth (PPD) was measured three times in each location using the Michigan “O” probe with Williams marking. The probe was inserted in the direction parallel to the tooth’s long axis. Six different locations were measured as follows: the mesio-buccal, mid-buccal, disto-buccal, mesio-lingual/palatal, mid-lingual/palatal, and disto-lingual/palatal areas of the tooth. Clinical examination was performed at days 0, 7, 14, 21, and 56 by the same examiner (PB).

### 2.5. Radiographic Examination

Intra-oral radiographs were used for assessment of alveolar bone loss with a Port-X II Portable Dental X-ray at 60 kV, 2 mA (iM3^®^, Vancouver, WA, USA). Alveolar bone loss was evaluated by direct measurement of the distance between the cemento-enamel junction (CEJ) and alveolar margin (AM) in relation to the root tooth length. The height of CEJ and AM were measured on the mesial and distal aspects of each tooth to quantify the alveolar bone level. Root length was measured from the CEJ to the root apex. Subsequently, the ratio of the distance from CEJ to the alveolar margin (CEJ/AM) and the distance of the CEJ to the outer aspect of the apex (CEJ/root apex) were calculated. Data were presented as a mean value for each tooth [29,30]. Radiographic examination was performed at days 0 and 56 by the same examiner (PB).

### 2.6. Histopathological Analysis

Histopathological analyses were performed to evaluate tissue inflammation and healing. Gingival tissues were collected from the buccal site of the tooth (size 3 × 5 mm) in accordance with Takahashi, Takashiba Takahashi et al. [31]. The tissue sampling was carried out at days 0, 14, and 56 under anesthesia. The samples were fixed with 10% neutral buffered formalin for 48 h, dehydrated in a graded series of ethanol solution, embedded in paraffin, and sectioned (5-μm thickness). Samples were stained with hematoxylin and eosin (H&E) and Masson’s trichrome to assess the inflammatory response and the healing process. The mean value was calculated in triplicate and recorded to the criteria described in Table 2 [32]. 

### 2.7. Gene Expression Analysis 

Gingival tissues were collected from the buccal site of the tooth using the methods of Takahashi et al. [31]. Gene expression analysis was performed at days 0, 7, and 14 to evaluate inflammatory cytokine- and growth factor-related gene expression. Tissues were then washed and homogenized in Trizol reagent (RiboEx Solution, GeneAll, Songpa-gu, South Korea) following the manufacturer’s instructions. The extracted RNA was treated with RNAse-free DNAse I (Ambion, Thermo Fisher Scientific, Waltham, MA, USA). The purity and quantity of RNA samples were evaluated using a Nanodrop spectrophotometer (Thermo Fisher Scientific, Waltham, MA, USA). Complementary DNA was synthesized using a cDNA reverse transcriptase kit (Promega, Madison, WI, USA) according to the manufacturer’s protocol. Real-time quantitative PCR was performed using FastStart^®^ Essential DNA Green Master (Roche Applied Science, Indianapolis, IN, USA) in a LightCycler96 (Roche Applied Science, Indianapolis, IN, USA). Post-amplification melting curve analysis was performed to evaluate product specificity. Expression values were calculated using the 2^−∆∆Ct^ method. *ACTB* expression levels were used as the reference. The oligonucleotide sequences are shown in Table 3.

### 2.8. Statistical Analyses

Non-parametric statistical analyses were employed in the present study. For three or more group comparisons, Friedman test was employed, followed by Dunn’s multiple comparison test. A Wilcoxon match-pairs signed-rank test was utilized for two group comparisons. Data analyses were performed using Prism8 (GraphPad Software, San Diego, CA, USA). A difference was considered significant when the *p* value < 0.05. 

## 3. Results

### 3.1. Clinical Examination

**Figure 2:**
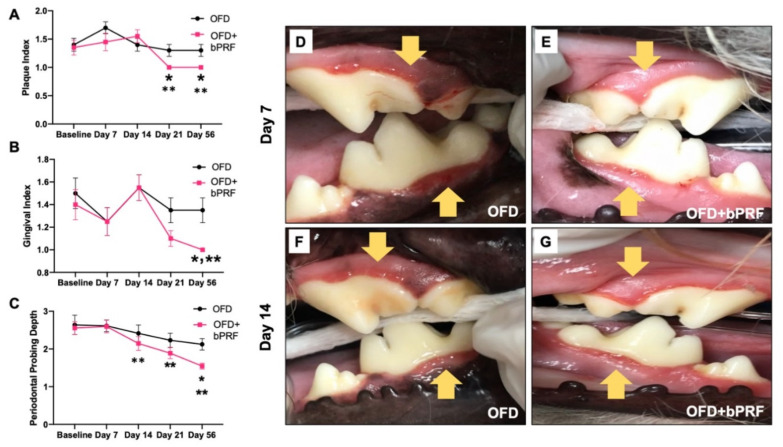
Bubaline blood-derived platelet-rich fibrin (bPRF) improved clinical periodontal parameters. Plaque index (**A**), gingival index (**B**), and periodontal pocket depth (**C**) were evaluated on days 0 (baseline), 7, 14, 21, and 56. Representative images of gingival status in the OFD and OFD+bPRF groups at day 7 (**D**,**E**) and day 14 (**F**,**G**) post operation. Yellow arrows indicate the surgical area. OFD; open-flap debridement-treated group, OFD+bPRF; open-flap debridement and bubaline blood-derived platelet-rich fibrin-treated group. Asterisks (*) indicate significant differences between OFD and OFD+bPRF groups at the same time point. Double asterisks (**) indicate significant differences compared with the baseline.

Both the open-flap debridement (OFD) and OFD with bPRF (OFD+bPRF) groups exhibited decreased scores in all clinical parameters, namely plaque index (PI), gingival index (GI), and periodontal probing depth (PPD), at later time points (Figure 2A–C). However, there were no statistically significant differences in all clinical parameters in the OFD group among observed time points. Defects treated with bPRF exhibited a significant decrease in PI at day 21 and 56 compared with the baseline (day 0) (Figure 2A). Further, bPRF treatment led to a significant decrease in GI at day 56, as well as a marked decrease in PPD at days 14, 21 and 56 compared with the baseline (Figure 2B,C). bPRF application resulted in a reduction in PI at days 21 and 56 compared with the OFD group (Figure 1A). A significant decrease in GI and PPD was observed at day 56 in the OFD+bPRF compared to the OFD group (Figure 2B,C). Representative images of the gingival status in the OFD and OFD+bPRF groups at day 7 and 14 after surgery are illustrated in Figure 2D–G. 

### 3.2. Radiographic Examination

**Figure 3:**
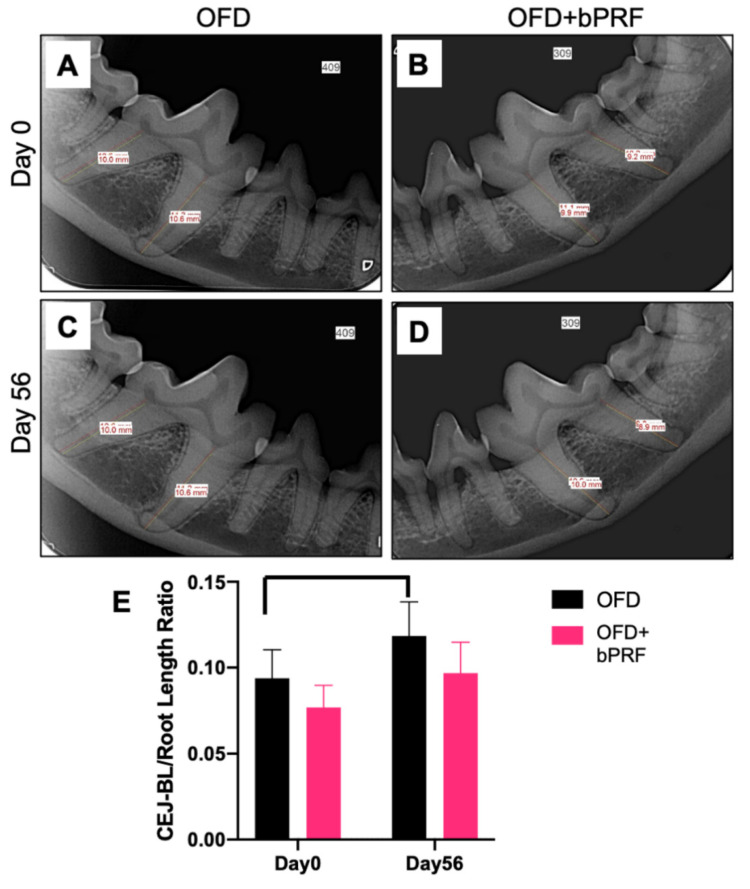
Bubaline blood-derived platelet-rich fibrin (bPRF) did not affect alveolar bone regeneration. Radiographic images were performed to evaluate alveolar bone regeneration. Representative radiographs demonstrated the levels of alveolar bone in the OFD and OFD+bPRF groups at baseline (**A**,**B**) and day 56 after intervention (**C**,**D**). The proportional distance between cemento-enamel junction (CEJ) and alveolar margin (AM) relative to root length was calculated (**E**). OFD; open-flap debridement-treated group, OFD+bPRF; open-flap debridement and bubaline blood-derived platelet-rich fibrin-treated group. Bars indicate a statistically significant difference.

Alveolar bone regeneration was evaluated at day 56 using radiographic analysis (Figure 3A–D). The cementoenamel junction-alveolar margin (CEJ-AM)/root length ratio was calculated from radiographs. The results demonstrated that the OFD+bPRF group exhibited a slight reduction in the CEJ-AM/root length ratio, but there was no statistically significant difference between the OFD and OFD+bPRF groups (Figure 3E). The OFD group showed a significant increase in the bone loss ratio at day 56 compared with day 0.

### 3.3. Histopathological Analyses

**Figure 4:**
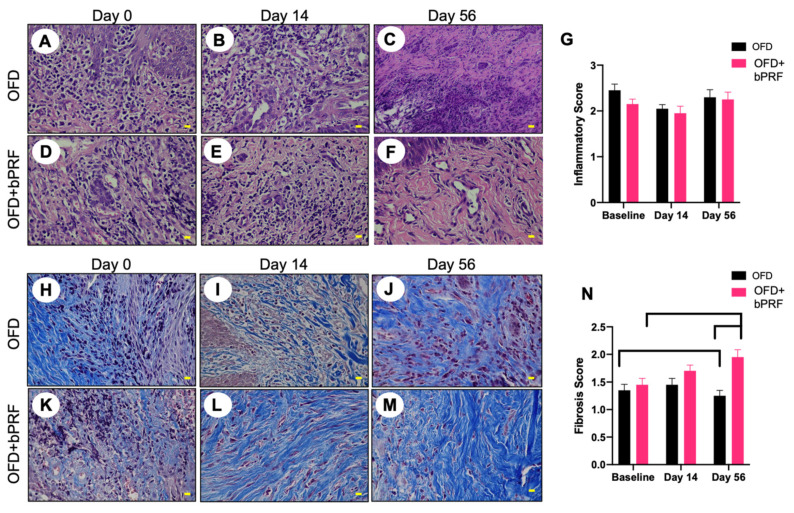
Bubaline blood-derived platelet-rich fibrin (bPRF) enhanced extracellular matrix production. Gingival tissues were collected from the buccal area of the tooth at days 0, 14, and 56 after intervention. Representative images of hematoxylin and eosin-stained (**A**–**F**) and Masson’s trichrome-stained (**H**–**M**) sections were demonstrated. Inflammatory and fibrosis scores were illustrated ((**G**,**N**), respectively). OFD; open-flap debridement-treated group, OFD+bPRF; open-flap debridement and bubaline blood-derived platelet-rich fibrin-treated group. Yellow scale bars indicate 10 µm. Bars indicate a statistically significant difference.

Histopathological observation indicated that OFD and OFD+bPRF groups exhibited an inflammatory cell infiltration, particularly lymphocytes and plasma cells (Figure 4A–F). The inflammatory scores were high in both groups at baseline (Figure 4G). Both the OFD and OFD+bPRF groups exhibited similar inflammatory scores at all time points. Connective tissue formation was examined using Masson’s trichrome staining. Results revealed the increased trends of fibrocyte and collagen accumulation in the OFD+bPRF group (Figure 4H–M). Organized collagen bundles were found in the OFD+bPRF group. At day 56, the OFD+bPRF group exhibited significantly higher fibrosis scores compared with the OFD group (Figure 4N). When compared with the baseline, the OFD group exhibited significantly decreased fibrosis scores, while a marked increase was observed in the OFD+bPRF group. 

### 3.4. Gene Expression Analysis

**Figure 5:**
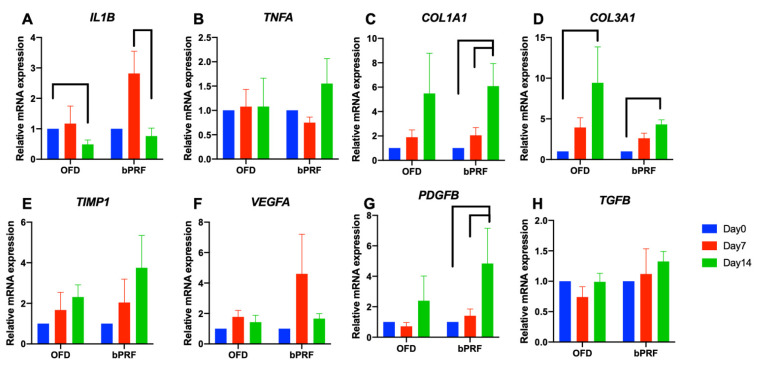
Gene expression analysis of gingival tissues from bubaline blood-derived platelet-rich fibrin (bPRF). Gingival tissues were collected from buccal site of the tooth at days 0, 7, and 14 after intervention. Total RNA was extracted, and the mRNA expression levels were examined using real-time polymerase chain reaction (**A**–**H**). OFD; open-flap debridement-treated group, OFD+bPRF; open-flap debridement and bubaline blood-derived platelet-rich fibrin-treated group. Bars indicate a statistically significant difference.

The effect of bPRF application on the inflammatory gene expressions, *IL1B* and *TNFA*, was evaluated. bPRF treatment resulted in an increase in *IL1B* mRNA expression at day 7 compared with the baseline (Figure 5A). However, a significant decrease in *IL1B* mRNA levels was observed at day 14. Expression of *TNFA* mRNA was similar at all time points in both OFD and OFD+bPRF groups (Figure 5B). Interestingly, bPRF application significantly increased *COL1A1* and *COL3A1* mRNA expression at day 14 compared with day 0 (Figure 5C,D). *COL1A1* mRNA levels slightly increased at day 14 compared with day 0 in the OFD group. However, there was no statistically significant difference. *TIMP1* mRNA expression showed an increased trend in both OFD and OFD+bPRF groups in a time-dependent manner but there was no statistically significant difference (Figure 5E). For expression of growth factor-related genes, bPRF treatment increased *VEGF* mRNA levels at day 14, but no significant difference was observed (Figure 5F), while *VEGF* mRNA levels were similar at all time points in OFD group. In the OFD+bPRF group, *PDGFB* mRNA expression was significantly upregulated at day 14 compared with day 0 and 7 (Figure 5G). There was no difference in *PDGFB* mRNA expression levels in the OFD group. Furthermore, *TGFB* mRNA levels were similar in both OFD and OFD+bPRF groups at all time points (Figure 5H).

## 4. Discussion

The present study described the use of platelet-rich fibrin xenologous membranes derived from bubaline blood in canine periodontal defects. A split-mouth design was employed in the present study. bPRF improved gingival index and periodontal probing depth. bPRF treatment upregulated genes related to growth factor at day 14. Hence, the results imply that bPRF application could be used to promote periodontal wound healing. However, the beneficial influence of bPRF on periodontal regeneration requires further investigation and more information to conclude.

Platelet concentrates and related products including PRF have now been highlighted in periodontal clinical application in companion dogs; our previous study confirmed the influence of autologous PRF in canine periodontal healing [19]. Regarding the concern of autologous health status and a limitation of a high-volume blood obtained for autologous PRF preparation in small dog breeds, the present study demonstrated that xenologous bPRF could be an alternative source of platelet concentrate for periodontitis treatment in small companion dogs. Bubaline blood-related products, such as bubaline fibrin glue, have been used as a bioactive surgical adhesive to promote skin graft survival, increasing the success of skin graft surgery in experimental pigs [33]. The present study is the first to report the utilization of xenologous bPRF as an adjuvant therapeutic agent for regenerative treatment in canine periodontal defects. bPRF application after OFD in stage 2 periodontitis in dogs resulted in a reduction in GI and PPD scores. Correspondingly, previous studies have demonstrated the beneficial effects of PRF in periodontal healing and/or regeneration [18,19,34,35]. Bovine porous bone materials (BPBM) in combination with PRF improved clinical parameters by reducing PPD in human intrabony periodontal defects [36]. These findings indicate the efficacy of PRF in periodontal regenerative treatment.

Histopathological observation in the present study showed an inflammatory reaction in both the OFD and OFD+bPRF groups at day 14 after operation. The OFD+bPRF group showed a lower, but not significant, necrotic area inflammatory response. This phenomenon could be due to the fact that bubaline blood exhibits positive regulation in immunological responses to host tissue. Moreover, bPRF contains a higher number of platelet entrapments. We hypothesized that the release of anti-inflammatory cytokines from bPRF would decrease the inflammatory reaction. The effect of bPRF on inflammation requires further investigation.

The present study indicated a significant increase in fibrosis score at day 56 in the OFD+bPRF group. Further, gene expression analyses revealed the significant increase in *COL1A1* and *COL3A1* mRNA expression in the bPRF-treated groups. Corresponding to previous studies, PRF enhanced *COL1A1* and *COL3A1* mRNA expression in rabbit tenocytes in vitro [37]. Type III collagen is predominant in the proliferative phase of wound healing and is replaced by type I collagen. Hence, all these observations accumulate evidence to support the effect of bPRF in the promotion of connective tissue formation in periodontal healing in dogs. The role of PRF in soft tissue healing has previously been reported. PRF accelerated epithelialization and lessened scar formation in canine cutaneous incisional wound healing [37]. PRF also stimulated collagen-related protein expression compared with untreated controls [38]. PRF could serve as a reservoir of bioactive molecules to stimulate tissue healing and regeneration. PRF contains several growth factors, including PDGF, TGF-B1, VEGF, IGF1, FGF, and EGF [39,40,41,42]. These factors are released gradually from PRF within 7 days [43] and facilitate wound healing mechanisms, for example, chemotaxis, inflammation, proliferation of granulation tissue, reepithelization, extracellular matrix formation, and remodeling [44]. 

PDGF has a crucial role in periodontal wound healing and regeneration. A systematic review in human studies reported that PDGF together with graft materials had a positive effect on periodontal defect healing, gingival recession treatment, sinus floor augmentation, and alveolar ridge preservation [45]. PDGF-BB promotes periodontal ligament cell proliferation and osteogenic differentiation in vitro [46]. It was also shown that PDGF-BB enhanced cell adhesion to the root surface in vitro [47]. The present study showed a marked increase in *PDGFB* mRNA expression at 14 days compared with day 0 and 7 in the OFD+bPRF group. Hence, *PDGF* expression is hypothesized to facilitate the improved clinical parameters observed in the bPRF treated group.

This study was the first animal-model study to investigate the efficacy of bubaline blood-derived PRF. New alternatives to the treatment of periodontal disease, particularly in small dogs, would help improve treatment outcomes and help prevent serious complications associated with advanced periodontal disease. Further studies should investigate the success of treating deep vertical pockets in small dog breeds with advanced periodontal disease using bubaline blood derived PRF to reduce periodontal pocket depth and to replace alveolar bone loss in small dog breeds with vertical bony pockets. Our previous study reported the excellent biological response of canine periodontal ligament cells on Thai and Murrah bubaline blood derived PRF in vitro [48]. However, there were some limitations of this study. The animal models with the early stage of periodontitis do not exhibit extensive suprabony defects. Hence, the results showed no significant levels of alveolar bone loss after intervention. The advanced stage of periodontitis should be further investigated to elucidate the potential mechanism of bubaline blood derived PRF in periodontal wound healing and regeneration. In addition, we employed the PRF membranes without serum in the surgical areas. Although the exudate serum was used to preserve and storage the PRF prior to placement, the amount of growth factors remained in the PRF could be reduced. Thus, the results should be interpreted with caution.

## 5. Conclusions

Taking all the evidence together, bPRF positively promotes periodontal wound healing in terms of increased collagen accumulation and induced growth factor expression. bPRF application improves clinical parameters including PI, GI, and PPD after periodontal defect treatment. Hence, bPRF could be an alternative biomaterial for the treatment of periodontal defects in small companion dogs. However, the influence of bPRF on periodontal regeneration requires further investigation and information.

## Figures and Tables

**Table 1:** Clinical parameter.

**Plaque Index**	
score 0	no plaque
score 1	less than 25% plaque accumulation on the free gingival margin and the surface of the tooth
score 2	25–50% plaque accumulation on the surface of the tooth and deposits in the periodontal pocket
score 3	more than 50% plaque accumulation on the gingival margin and abundant plaque in the periodontal pocket
**Gingival Index**	
score 0	no inflammation and healthy periodontium
score 1	mild inflammation with slight change in the color of the gingiva (erythyma), rounding of the gingival edges, and no bleeding on probing
score 2	moderate inflammation with a significant change in the color (redness), rounding of the gingival edges, as well as bleeding on probing
score 3	severe inflammation with a significant change in the color (redness), the consistency (hypertrophy), and the density of the gingiva (ulceration), as well as spontaneous bleeding

**Table 2:** Histopathological score.

Inflammatory Response Score	
Score 0	no inflammatory cells
Score 1	inflammatory cells found 1–35% of the field
Score 2	inflammatory cells found in 36–70% of the fields
Score 3	inflammatory cells found over 70% of the fields
Healing Response Score	
Score 0	no fibroblasts, fibrocytes, or connective tissues
Score 1	fibroblasts, fibrocytes, and connective tissues found 1–35% of the fields
Score 2	fibroblasts, fibrocytes, and connective tissues found 36–70% of the fields
Score 3	fibroblasts, fibrocytes, and connective tissues found over 70% of the fields

**Table 3:** Oligonucleotide sequences.

Primer	Accession Number	Forward Primer	Reverse Primer
*ACTB*	NM_001195845.2	5′-AGCTCCACGGAGAAGAACTG-3′	5′-GGCTCCAAATGTAGGGGCAG-3′
*TNFA*	NM_001003244	5′-TCTCGAACCCCAAGTGACAAG-3′	5′-CAACCCATCTGACGGCACTA-3′
*PDGFB*	NM_001003383.1	5′-ACCGGAAGTTCAAGCACACA-3′	5′-TGCCCTCAATCTCCTCCAGA-3′
*TGFB1*	NM_001003309.1	5′-GGACTTCGAGCAGGAGATGG-3′	5′-TTCCATGCCCAGGAAGGAAG-3′
*VEGFA*	NM_001003175.2	5′-CCGGTATAAACCCTGGAGCG-3′	5′-GCAACGCGAGTCTGTGTTTT-3′
*IL1B*	NM_001037971	5′-CAAGTCTCCCACCAGCTCTGTA-3′	5′-GGGCTTCTTCAGCTTCTCCAA-3′
*COL1A1*	AF153062.1	5′-GGCAGGAGGGTTCAGCTAAG-3′	5′-GCAACAAAGTCCGCGTATCC-3′
*COL3A1*	HM775210.1	5′-TTCCTGGGAGAAATGGCGAC-3′	5′-AGGACCAGTAGGGCAGGATT-3′
*TIMP1*	AF077817_1	5′-GATGTTCAAGGGTTTCAGCG-3′	5′-TGTCACTCTGCAGTTGCAG-3′

## Data Availability

Not applicable.
